# Epitope Shaving Promotes Fungal Immune Evasion

**DOI:** 10.1128/mBio.00984-20

**Published:** 2020-07-07

**Authors:** Delma S. Childers, Gabriela Mol Avelar, Judith M. Bain, Arnab Pradhan, Daniel E. Larcombe, Mihai G. Netea, Lars P. Erwig, Neil A. R. Gow, Alistair J. P. Brown

**Affiliations:** a Aberdeen Fungal Group, Institute of Medical Sciences, University of Aberdeen, Aberdeen, United Kingdom; b Medical Research Council Centre for Medical Mycology, School of Biosciences, University of Exeter, Exeter, United Kingdom; c Department of Internal Medicine and Radboud Center for Infectious Diseases, Radboud University Medical Center, Nijmegen, Netherlands; d Department for Immunology & Metabolism, Life and Medical Sciences Institute (LIMES), University of Bonn, Bonn, Germany; e Experimental Medicine, Galvani Bioelectronics, Stevenage, United Kingdom; University of Texas Health Science Center

**Keywords:** *Candida albicans*, cell wall, Xog1 exoglucanase, β-glucan shaving, immune evasion, castanospermine, virulence

## Abstract

The cell wall provides a major physical interface between fungal pathogens and their mammalian host. This extracellular armor is critical for fungal cell homeostasis and survival. Fungus-specific cell wall moieties, such as β-1,3-glucan, are recognized as pathogen-associated molecular patterns (PAMPs) that activate immune-mediated clearance mechanisms. We have reported that the opportunistic human fungal pathogen Candida albicans masks β-1,3-glucan following exposure to lactate, hypoxia, or iron depletion. However, the precise mechanism(s) by which C. albicans masks β-1,3-glucan has remained obscure. Here, we identify a secreted exoglucanase, Xog1, that is induced in response to lactate or hypoxia. Xog1 functions downstream of the lactate-induced β-glucan “masking” pathway to promote β-1,3-glucan “shaving.” Inactivation of *XOG1* blocks most but not all β-1,3-glucan masking in response to lactate, suggesting that other activities contribute to this phenomenon. Nevertheless, *XOG1* deletion attenuates the lactate-induced reductions in phagocytosis and cytokine stimulation normally observed for wild-type cells. We also demonstrate that the pharmacological inhibition of exoglucanases undermines β-glucan shaving, enhances the immune visibility of the fungus, and attenuates its virulence. Our study establishes a new mechanism underlying environmentally induced PAMP remodeling that can be manipulated pharmacologically to influence immune recognition and infection outcomes.

## Abstract (precis)

The immune system plays a critical role in protecting us against potentially fatal fungal infections. However, some fungal pathogens have evolved evasion strategies that reduce the efficacy of our immune defenses. Previously, we reported that the fungal pathogen Candida albicans exploits specific host-derived signals (such as lactate and hypoxia) to trigger an immune evasion strategy that involves reducing the exposure of β-glucan at its cell surface. Here, we show that this phenomenon is mediated by the induction of a major secreted exoglucanase (Xog1) by the fungus in response to these host signals. Inactivating *XOG1*-mediated “shaving” of cell surface-exposed β-glucan enhances immune responses against the fungus. Furthermore, inhibiting exoglucanase activity pharmacologically attenuates C. albicans virulence. In addition to revealing the mechanism underlying a key immune evasion strategy in a major fungal pathogen of humans, our work highlights the potential therapeutic value of drugs that block fungal immune evasion.

## INTRODUCTION

Life-threatening fungal infection outcomes are affected by the efficacy of the immune system in recognizing and clearing potential invaders (1). Fungal diseases cause more than 1 million deaths worldwide annually (2). *Candida* species are a leading cause of life-threatening fungal infections, and Candida albicans is the species responsible for the majority of these *Candida* infections (2). C. albicans is an obligate commensal of warm-blooded animals that causes opportunistic mucosal and systemic infections in immunocompromised patients and other susceptible populations, including individuals requiring abdominal surgery or long-term intensive care (3). The success of C. albicans as a fungal pathogen is widely attributed to virulence factors such as its ability to undergo a reversible morphological transition between yeast and filamentous cells, the production of adhesins, secreted proteases and candidalysin, and the ability to evade immune killing (4–6).

Host clearance mechanisms are initiated via pathogen-associated molecular pattern (PAMP) recognition by pattern recognition receptors (PRRs) (7). The fungal cell wall is the primary point of direct contact with host cells, and it plays a key role in immune recognition (8). Fungal cell walls contain major PAMPs that can induce inflammatory or tolerogenic processes (7, 9). β-1,3-Glucan, in particular, is highly inflammatory. In C. albicans, most β-1,3-glucan is located in the inner cell wall and is masked from immune recognition by the outer layer of mannan fibrils (10). When β-1,3-glucan becomes exposed at the cell surface, it is recognized by the C-type lectin dectin-1 (11), and this PAMP-PRR interaction plays a key role in antifungal immunity (11–14).

C. albicans presents a moving immunological target to the host. For example, C. albicans cells reduce the exposure of β-1,3-glucan at their cell surface in response to specific host signals. When C. albicans is exposed to l-lactate, a metabolite generated by cells of the host and its microbiota, the fungus activates signaling via the conserved G-protein coupled receptor (GPCR), Gpr1, and the transcription factor, Crz1 (15). This culminates in decreased β-1,3 glucan exposure and impacts host immune responses by reducing phagocytosis and cytokine production by innate immune cells. Additional host-relevant conditions, including hypoxia, shifts in ambient pH, and iron starvation, influence cell wall architecture and PAMP exposure (16–18). However, the mechanisms governing β-1,3 glucan masking in C. albicans have proven elusive.

In this study, we utilized proteomics to identify a secreted exo-β-1,3-glucanase, Xog1, that acts in *trans* to mediate lactate-induced β-glucan masking in C. albicans. We show that deletion of *XOG1*, or pharmacological inhibition of exoglucanase, inhibits lactate-induced β-glucan masking and results in a higher phagocytic uptake by macrophages and cytokine responses of human peripheral blood mononuclear cells (PBMCs) compared to their wild-type or untreated controls, respectively. Furthermore, we demonstrate that inhibition of exoglucanase activity attenuates the virulence of C. albicans in a Galleria mellonella model of systemic infection. Taken together, our data suggest that this major fungal pathogen evades immune recognition and promotes infection by shaving a major cell surface PAMP.

## RESULTS

### Extracellular factors mediate β-glucan masking.

We reasoned that β-glucan masking might be mediated by proteins that are secreted by C. albicans into the milieu and, if so, these proteins might act in *trans* to mask β-glucan on masking-defective cells. To test this, we investigated whether culture supernatants from wild-type cells could functionally complement the defect in lactate-induced β-glucan masking displayed by a masking-deficient *czf1Δ* mutant.

**FIG 1:**
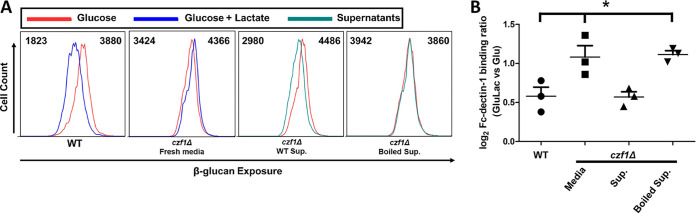
An extracellular factor is required for lactate-induced β-glucan masking. (A) C. albicans wild-type (WT) SC5314 cells were grown in glucose alone or glucose plus lactate, while *czf1*Δ cells were grown in glucose alone and then treated with boiled or untreated culture supernatants from wild-type cells grown on glucose plus lactate. The β-glucan exposure on these cells was assayed by Fc-dectin-1 staining and flow cytometry. Left to right, respectively: wild-type cells, glucose versus glucose plus lactate; *czf1*Δ cells, glucose +/− fresh medium; *czf1*Δ cells, glucose +/− wild-type culture supernatant; *czf1*Δ cells, glucose +/− boiled wild-type culture supernatant. The median fluorescence intensity for each population is indicated. (B) Fold changes in β-glucan exposure for this experiment are presented relative to glucose-grown control cells. *n* = 3; *, *P* < 0.05 (one-way ANOVA with Tukey’s *post hoc*).

*CZF1* is a zinc-finger transcription factor that regulates C. albicans contact-dependent morphogenesis and white-opaque switching (19, 20), and we have reported that *czf1*Δ cells display a defect in lactate-induced β-glucan masking (15). Therefore, *czf1*Δ cells were incubated for 4 h with fresh lactate-containing medium, spent medium from congenic wild-type C. albicans cells (SC5314), or the same spent medium that had been boiled and cooled. Their degree of β-glucan exposure was quantified by staining cells with Fc-dectin-1 and analyzing them by flow cytometry. We compared them to control cells grown on medium lacking lactate. As expected (15), control wild-type C. albicans cells displayed β-glucan in response to lactate, but *czf1*Δ cells did not (Fig. 1). However, when the *czf1*Δ cells were incubated with spent medium from wild-type cells, β-glucan masking was restored. This effect was abrogated if the spent medium had been boiled (Fig. 1), suggesting that heat-denaturable factors in wild-type supernatants are sufficient to rescue β-glucan masking in a masking-deficient mutant.

### Proteomic fingerprints of β-glucan masking.

The factors involved in β-glucan masking might be proteins, and C. albicans expresses genes encoding cell wall-anchored or secreted carbohydrate remodeling enzymes that could mediate β-glucan masking. However, our genome-wide transcriptomic comparisons of lactate-exposed versus control C. albicans cells did not highlight differentially expressed cell wall genes that might contribute to β-glucan masking (15). Therefore, we reasoned that such proteins might be subject to posttranscriptional and/or posttranslational regulation. To address this, we performed unbiased liquid chromatography-tandem mass spectrometry (LC-MS/MS) analysis of the cell wall proteomes and secretomes for control C. albicans SC5314 cells and compared them to cells undergoing lactate- or hypoxia-induced β-glucan masking.

**FIG 2:**
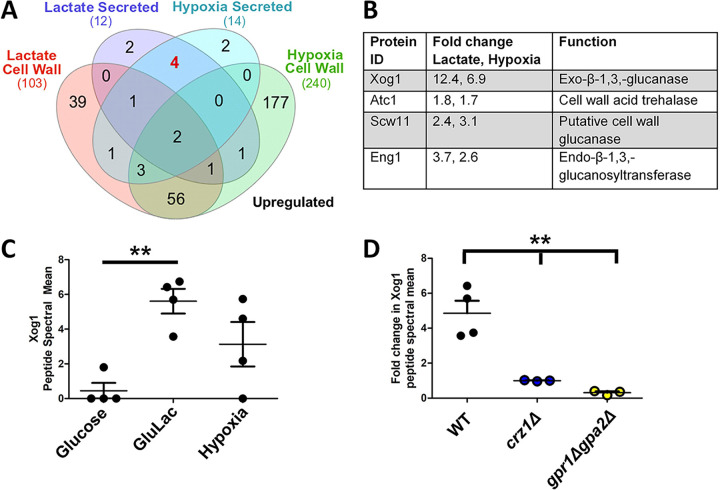
The Xog1 protein and exoglucanase activity are upregulated during β-glucan masking. (A) Proteomic analysis of cell walls and secretomes of cells grown in lactate versus the glucose-grown control, and hypoxic cells versus the aerobic-grown control. Venn diagram of proteins displaying >1.5-fold upregulation in peptide spectral mean (PSM) relative to the control. *n* = 4. (B) Table of extracellular proteins displaying >1.5-fold upregulation in PSM in lactate-grown or hypoxia-grown cells compared to their respective controls. *n* = 4. (C) Peptide spectral means for Xog1 from the supernatants of C. albicans cells grown in glucose, glucose plus lactate, and hypoxic conditions. *n* = 4. (D) Fold change in peptide spectral means for Xog1 in wild-type, *crz1*Δ, and *gpr1*Δ *gpa2*Δ cells grown in glucose plus lactate compared to glucose alone. *n* = 3 to 4. **, *P* < 0.01, one-way ANOVA with Tukey's *post hoc*.

We identified 213 proteins in the cell wall proteome that were differentially regulated under lactate-induced β-glucan masking conditions, and 392 differentially regulated proteins under hypoxic conditions compared to glucose and normoxic controls (Fig. 2A; see also Fig. S1 in the supplemental material). Fewer proteins were recovered from the secretome compared to the cell wall proteome, and fewer proteins were differentially regulated in the secretome (*n* = 79 in total for the lactate and hypoxia treatments compared to the control) compared to the cell wall proteome (*n* = 526).

10.1128/mBio.00984-20.1 FIG S1 Hydrolase and glucosidase activities are represented at higher frequency in β-glucan masking cells compared to glucose-grown cells. (A and B) GO molecular function analysis was performed for upregulated peptides under β-glucan masking conditions compared to glucose-grown controls from cell wall proteomics (A) and secretomics (B). (C) Venn diagram of proteins with <0.5-fold change in peptide spectral mean (PSM) from cells grown in lactate or hypoxia compared to glucose. *n* = 4. Download FIG S1, PDF file, 0.5 MB. Copyright © 2020 Childers et al. 2020 Childers et al. This content is distributed under the terms of the Creative Commons Attribution 4.0 International license.

We surmised that proteins involved in β-glucan masking are likely to be under positive regulation, and therefore more abundant under masking conditions. Thus, we focused on the proteins that were induced in response to lactate and/or hypoxia compared to glucose-grown cells. GO molecular function analyses of the lactate- and hypoxia-induced proteins (Fig. S1) revealed that hydrolases with activity toward glycosyl bonds were enriched in both lactate and hypoxic cell wall proteomes and secretomes. Strikingly, the lactate- and hypoxia-induced secretomes displayed enrichment for proteins with glucosidase and glucan exo-1,3-β-glucosidase activity. Indeed, three of the four secreted proteins that were induced by both lactate and hypoxia have characterized or putative glucanase activity (Fig. 2B). Dectin-1 is known to bind β-glucans with chain lengths of 10 or more glucose units (21). This was consistent with the idea that β-glucan “masking” may be driven by glucanase-mediated truncation of β-glucan chain lengths to reduce dectin-1 targets in the cell wall.

### The major exoglucanase, Xog1, plays a key role in β-glucan masking.

C. albicans encodes three exoglucanases: Xog1, Exg2, and Spr1. *EXG2* is induced in response to lactate, but *exg2*Δ cells display only a mild β-glucan masking defect (15). Also, our proteomics experiments provided no evidence of the Spr1 protein under the conditions examined. Gonzalez and coworkers (22) demonstrated that *XOG1* is responsible for the majority of measurable exoglucanase activity in C. albicans. We found that Xog1 was the protein that displayed the greatest fold change in abundance in the secretomes of lactate- and hypoxia-exposed cells (Fig. 2B). Thus, we set out to determine whether Xog1 plays a role in lactate-induced β-glucan masking.

Our previous transcript profiling study suggested that *XOG1* is not differentially expressed in response to lactate in wild-type or *crz1*Δ cells (15). To test whether the Xog1 protein is upregulated, we examined Xog1 peptide levels in the secretomes of wild-type C. albicans SC5314 cells (Fig. 2C). Xog1 peptide levels were significantly increased under lactate-induced β-glucan masking conditions, and Xog1 peptide was detected at higher levels under hypoxia than control conditions. This suggested that Xog1 levels are regulated at a posttranscriptional and/or posttranslational level. Furthermore, it was consistent with the idea that Xog1 promotes lactate- and hypoxia-induced β-glucan masking.

C. albicans *crz1*Δ and *gpr1*Δ *gpa2*Δ mutants are defective in β-glucan masking (15). Therefore, using LC-MS/MS, we examined the secretomes of *crz1*Δ and *gpr1*Δ *gpa2*Δ cells in the presence or absence of lactate. Both *crz1*Δ and *gpr1*Δ *gpa2*Δ cells displayed significant defects in Xog1 induction in response to lactate (Fig. 2D). Thus, the induction of Xog1 secretion appears to depend on the same signaling mechanisms that drive lactate-induced β-glucan masking.

**FIG 3:**
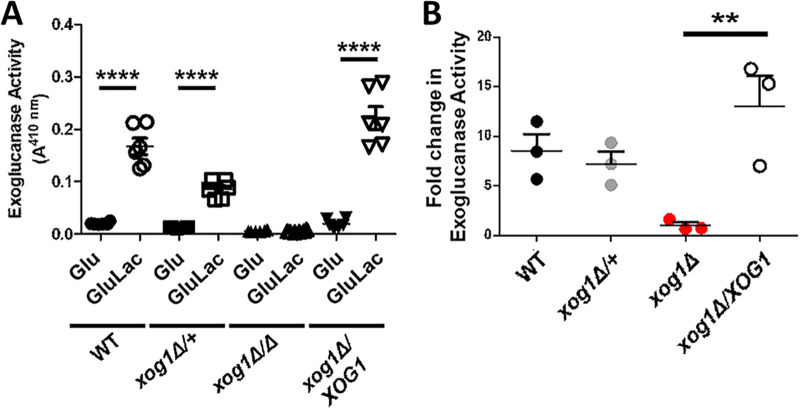
Inactivating Xog1 reduces exoglucanase activity and blocks its induction during lactate-induced β-glucan masking. (A) Exoglucanase activity was measured by monitoring PNPG cleavage in the indicated strains grown in glucose or glucose plus lactate medium. Experiments were performed in biological triplicate and technical duplicate. *n* = 3. **, *P* < 0.01; ****, *P* < 0.0001, two-way ANOVA with Tukey’s *post hoc*. (B) Fold change in extracellular exoglucanase activity for an isogenic set of C. albicans *xog1* strains grown in medium containing glucose or glucose plus lactate: wild type (*XOG1/XOG1*), heterozygote (*XOG1/xog1*Δ), homozygous null mutant (*xog1*Δ*/xog1*Δ), reintegrant (*xog1*Δ*/xog1*Δ*/XOG1*). *n* = 3. **, *P* < 0.01, one-way ANOVA with Tukey’s *post hoc*.

Next, we tested the impact of Xog1 inactivation upon exoglucanase levels and β-glucan masking. We sequentially deleted both alleles of *XOG1* in the wild-type C. albicans clinical isolate SC5314 to generate heterozygous (*xog1*Δ/+) and homozygous (*xog1*Δ) mutants (Table S1). We then reintroduced *XOG1* into the null mutant to generate a control reintegrant strain (*xog1*Δ*/XOG1*). We measured exoglucanase levels in culture supernatants for this isogenic strain set in the presence and absence of lactate. In agreement with previous studies (22, 23), we found that *XOG1* encodes the majority of extracellular exoglucanase activity for C. albicans (Fig. 3A). Furthermore, unlike the wild-type and reintegrant controls, *xog1*Δ cells did not show an increase in extracellular exoglucanase in response to lactate (Fig. 3B).

10.1128/mBio.00984-20.3 TABLE S1 Strains and oligonucleotides used in this study. Download Table S1, PDF file, 0.2 MB. Copyright © 2020 Childers et al. 2020 Childers et al. This content is distributed under the terms of the Creative Commons Attribution 4.0 International license.

**FIG 4:**
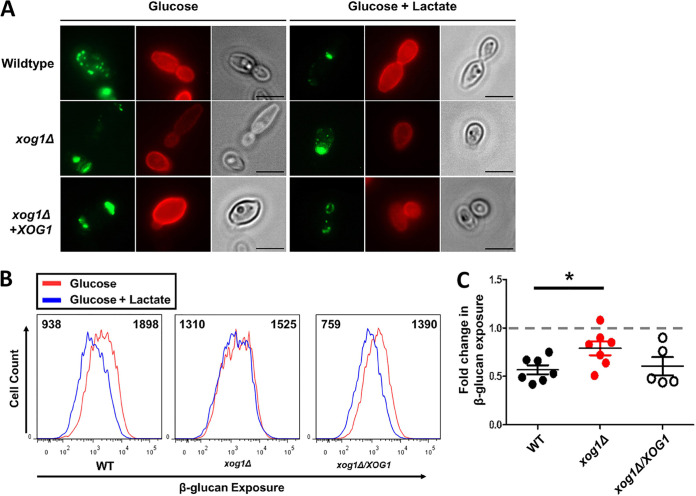
Inactivation of Xog1 inhibits lactate-induced β-glucan masking. (A) Microscopic analysis of β-glucan exposure in C. albicans cells grown in glucose or glucose plus lactate medium: wild type (*XOG1/XOG1*), homozygous null mutant (*xog1*Δ*/xog1*Δ), reintegrant (*xog1*Δ*/xog1*Δ*/XOG1*). Images representative of three biological replicate experiments. Cells were stained with Fc-dectin-1 for β-glucan exposure (green) and concanavalin A for mannan exposure (red); scale bar, 5 μm. (B) Flow cytometry plots of β-glucan exposure for the indicated strains grown in glucose (red) or glucose plus lactate (blue) from one representative biological sample of *n* = 3 independent replicates. The median fluorescence intensity for each population is indicated. (C) Fold change in Fc-dectin-1 binding for glucose plus lactate- versus glucose-grown cells, calculated from experiments such as those shown in panel B. *n* = 5 to 7. Statistical analyses: one-way ANOVA with Tukey’s *post hoc*. *, *P* < 0.05.

We then tested whether *XOG1* contributes to lactate-induced β-glucan masking. *XOG1* deletion does not affect cell wall architecture significantly (22). Nevertheless, microscopic analyses of dectin-1-stained cells showed that *xog1*Δ cells displayed a significant defect in lactate-induced β-glucan masking, compared to the wild-type control (Fig. 4A). Also, this masking defect was suppressed in the reintegrant strain. Bright Fc-dectin-1 staining was observed around bud/birth scars as well as punctate foci of β-glucan exposure on the rest of the C. albicans cell surface. Following lactate exposure, both types of feature were less intense in wild-type *XOG1* cells, but this decrease in intensity was not observed for *xog1*Δ cells (Fig. 4A). These microscopic observations were then confirmed by cytometric quantification of β-glucan exposure in cell populations. In contrast to the wild-type (*XOG1*) and reintegrant (*xog1*Δ*/XOG1*) strains, *xog1*Δ cells displayed a significant defect in lactate-induced β-glucan masking (Fig. 4B and C). Nevertheless, some masking remains, suggesting that, while Xog1 is the major exoglucanase under the conditions tested (Fig. 3A), other activities might contribute to lactate-induced reductions in β-glucan exposure.

### Xog1-mediated masking influences phagocytic interactions and cytokine responses.

The changes in β-glucan exposure that accompany adaptation to host-related environmental inputs often result in altered host-fungus interactions (15–18). Thus, we tested whether Xog1 influences host recognition of C. albicans cells and their interaction with host innate immune cells.

**FIG 5:**
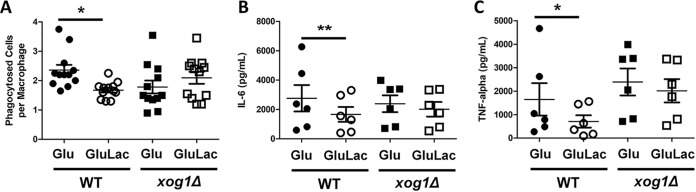
Exoglucanase-induced β-glucan masking impacts macrophage phagocytosis and cytokine elicitation. (A) C. albicans strains were grown in medium containing glucose or glucose plus lactate, inactivated in thimerosal, and coincubated with murine BMDMs: wild type (*XOG1/XOG1*), homozygous null mutant (*xog1*Δ*/xog1*Δ). Mean number of C. albicans cells phagocytosed per murine BMDM in 2 h; *n* = 4 mice analyzed in biological triplicate; >200 macrophages counted per coincubation. *, *P* < 0.05, one-way ANOVA with Tukey’s *post hoc*. (B and C) The same C. albicans strains, grown in glucose or glucose plus lactate and inactivated in thimerosal, were coincubated with human PBMCs. IL-6 (B) and TNF-α (C) levels were assayed after 24 h. *n* = 3 healthy donors, measured in duplicate (*n* = 3); *, *P* < 0.05; **, *P* < 0.01, two-way ANOVA with Tukey’s *post hoc*.

As predicted elsewhere (15), lactate-exposed wild-type C. albicans cells were phagocytosed less frequently by murine bone marrow-derived macrophages (BMDMs) than untreated control cells (Fig. 5A; Movies S1 and S2). Also following lactate exposure, wild-type cells elicited weaker cytokine responses from human PBMCs, which produced significantly lower levels of the proinflammatory cytokines IL-6 and TNF-α (Fig. 5B and C). In contrast, *xog1*Δ cells did not display significant reductions in phagocytic uptake (Fig. 5A; Movies S3 and S4) or attenuated cytokine responses (Fig. 5B and C) following lactate exposure. Both phenotypes were restored in *xog1*Δ*/XOG1* reintegrant cells. These results suggest that Xog1-mediated β-glucan remodeling plays an important role in C. albicans*-*host interactions. However, the inactivation of *XOG1* did not significantly reduce the virulence of C. albicans in Galleria mellonella (Fig. S2), which was consistent with findings in the mouse model of systemic candidiasis (22).

10.1128/mBio.00984-20.2 FIG S2 *XOG1* inactivation does not significantly attenuate C. albicans virulence. Percent survival of G. mellonella larvae infected with wild-type or *xog1Δ* C. albicans cells (*n* = 10). Download FIG S2, PDF file, 0.1 MB. Copyright © 2020 Childers et al. 2020 Childers et al. This content is distributed under the terms of the Creative Commons Attribution 4.0 International license.

10.1128/mBio.00984-20.5 MOVIE S1 First time-lapse video of BMDM interactions with glucose-grown C. albicans cells. This movie shows the first 2 h of interactions between murine BMDMs and wild-type C. albicans SC5314 cells. It is representative of 12 movies in total (4 movies from 3 mice). Download Movie S1, AVI file, 18.4 MB. Copyright © 2020 Childers et al. 2020 Childers et al. This content is distributed under the terms of the Creative Commons Attribution 4.0 International license.

10.1128/mBio.00984-20.6 MOVIE S2 Second time-lapse video of BMDM interactions with glucose-plus-lactate-grown C. albicans cells. This movie shows the first 2 h of interactions between murine BMDMs and wild-type C. albicans SC5314 cells. It is representative of 12 movies in total (4 movies from 3 mice). Download Movie S2, AVI file, 18.4 MB. Copyright © 2020 Childers et al. 2020 Childers et al. This content is distributed under the terms of the Creative Commons Attribution 4.0 International license.

10.1128/mBio.00984-20.7 MOVIE S3 First time-lapse video of BMDM interactions with C. albicans *xog1*Δ cells grown on glucose. This movie shows the first 2 h of interactions between murine BMDMs and C. albicans *xog1*Δ cells. It is representative of 12 movies in total (4 movies from 3 mice). Download Movie S3, AVI file, 18.4 MB. Copyright © 2020 Childers et al. 2020 Childers et al. This content is distributed under the terms of the Creative Commons Attribution 4.0 International license.

### Exoglucanase inhibition affects β-glucan masking and immune responses.

Our results thus far demonstrate that the *XOG1* gene promotes lactate-induced β-glucan masking and influences immune responses to C. albicans. Next, we confirmed that exoglucanase activity is required for these phenotypes.

**FIG 6:**
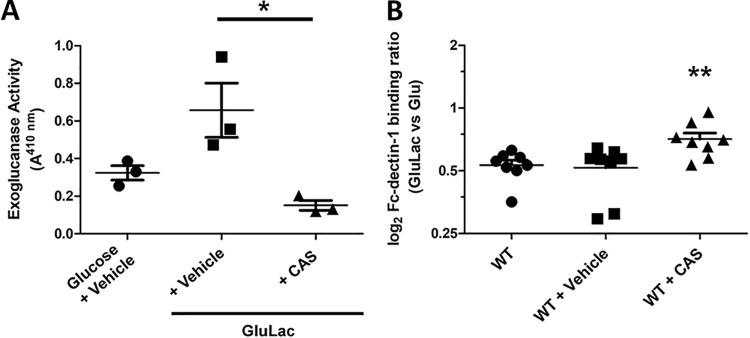
Castanospermine inhibits exoglucanase activity and β-glucan masking. (A) The effect of 1 μM castanospermine (CAS) upon the extracellular exoglucanase activity expressed by wild-type C. albicans cells grown in glucose or glucose plus lactate was determined using the PNPG assay. *n* = 3; *, *P* < 0.05, one-way ANOVA with Tukey’s *post hoc*. (B) The effect of 1 μM castanospermine on the change in β-glucan exposure observed for wild-type C. albicans cells grown in glucose plus lactate versus glucose: no addition, wild type (WT); DMSO, WT + vehicle; castanospermine, WT + 1 μM CAS. *n* = 8. *, *P* < 0.05; **, *P* < 0.01, one-way ANOVA with Tukey’s *post hoc*.

Castanospermine is the most potent inhibitor of Saccharomyces cerevisiae and C. albicans exoglucanase activity characterized to date (24). To confirm that castanospermine inhibits exoglucanase under our experimental conditions, we assayed the effects of castanospermine upon the extracellular exoglucanase generated by wild-type C. albicans cells using the chromogenic substrate 4-nitrophenyl-β-d-glucopyranoside (PNPG). As expected (24), castanospermine significantly attenuated extracellular exoglucanase activity (Fig. 6A).

We then tested whether castanospermine affects lactate-induced β-glucan masking in C. albicans. Control cells treated with vehicle alone displayed the normal reduction in β-glucan exposure in response to lactate (Fig. 6B). However, cells treated with castanospermine displayed a significant defect in lactate-induced β-glucan masking compared to the controls, suggesting exoglucanase plays a key role in remodeling β-glucan exposure in response to lactate.

**FIG 7:**
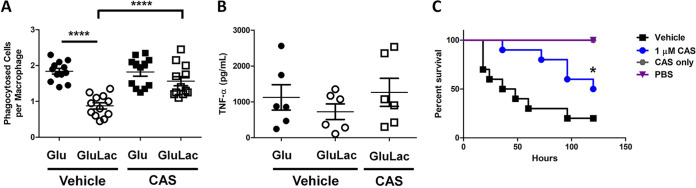
Inhibition of exoglucanase influences macrophage interactions, cytokine production, and virulence. (A) Mean number of wild-type C. albicans SC5314 cells phagocytosed per murine BMDM in 2 h: DMSO added, vehicle; 1 μM castanospermine added, CAS. *n* = 4 mice analyzed in biological triplicate, >200 macrophages counted per coincubation. ****, *P* < 0.0001, one-way ANOVA with Tukey’s *post hoc*. (B) Wild-type C. albicans cells were grown in glucose or glucose plus lactate, with or without vehicle or castanospermine (CAS), and fixed. TNF-α production was assayed after the fixed C. albicans cells were incubated with human PBMCs for 24 h. *n* = 3 healthy donors, measured in duplicate. (C) Percent survival of G. mellonella infected with wild-type C. albicans and treated with vehicle alone or 1 μM castanospermine. *n* = 10; *, *P* < 0.05, Kruskal-Wallis log rank test.

The impact of castanospermine treatment upon immune responses was then tested. Normally, exposing C. albicans to lactate reduces the ability of BMDMs to phagocytose the fungus and attenuates the cytokine responses of PBMCs (Fig. 5). However, castanospermine abrogated this reduction in BMDM phagocytosis (Fig. 7A; Movies S5 and S6) as well as the elicitation of TNF-α by PBMCs (Fig. 7B). Thus, either deleting *XOG1* or inhibiting exoglucanase significantly attenuates lactate-induced β-glucan remodeling and innate immune evasion.

10.1128/mBio.00984-20.8 MOVIE S4 Second time-lapse video of BMDM interactions with C. albicans *xog1Δ* cells grown on glucose plus lactate. This movie shows the first 2 h of interactions between murine BMDMs and C. albicans *xog1*Δ cells. It is representative of 12 movies in total (4 movies from 3 mice). Download Movie S4, AVI file, 18.4 MB. Copyright © 2020 Childers et al. 2020 Childers et al. This content is distributed under the terms of the Creative Commons Attribution 4.0 International license.

10.1128/mBio.00984-20.9 MOVIE S5 **F**irst time-lapse video of BMDM interactions with C. albicans cells grown on glucose plus lactate in the presence of castanospermine. This movie shows the first 2 h of interactions between murine BMDMs and wild-type C. albicans SC5314 cells. It is representative of 12 movies in total (4 movies from 3 mice). Download Movie S5, AVI file, 18.4 MB. Copyright © 2020 Childers et al. 2020 Childers et al. This content is distributed under the terms of the Creative Commons Attribution 4.0 International license.

### Exoglucanase inhibition attenuates virulence.

Next, we tested whether exoglucanase inhibition affects virulence outcomes during systemic candidiasis. Castanospermine is toxic in mice, inducing both weight loss and lethargy (25, 26), thereby precluding us from using this drug in the murine model of systemic candidiasis. Consequently, we used the G. mellonella invertebrate infection model, the outcomes of which correlate well with mouse survival studies (27). G. mellonella larvae died rapidly if they were infected with wild-type C. albicans cells and treated with vehicle only (Fig. 7C). However, infected larvae that were treated postinoculation with one dose of castanospermine displayed significantly extended survival times and increased survival rates (Fig. 7C). Larvae treated with castanospermine alone or with saline survived for the duration of the experiment. Therefore, castanospermine treatment extends host survival during a lethal challenge with C. albicans. This does not appear to be due to a loss of fitness, because *XOG1* inactivation or treatment with castanospermine does not inhibit yeast growth (22, 24), and exoglucanase activity is not essential for fitness *in vitro* (23). Therefore, castanospermine treatment probably promotes fungal clearance in the host.

## DISCUSSION

The exposure of the proinflammatory PAMP, β-1,3-glucan, drives phagocytic uptake and the release of proinflammatory cytokines, thereby promoting fungal killing and clearance from host tissues (8). There is no correlation between the thickness of the inner β-glucan-containing layer of the C. albicans cell wall and the degree of β-glucan exposure at the cell surface: hypoxia and iron limitation are both strong triggers of β-glucan masking and yet the former leads to a thin cell wall, while the latter leads to a thick cell wall (16, 17). Rather, the prevailing paradigm has been that most β-glucan in the inner cell wall of C. albicans is masked by the mannan in the outer layer of the cell wall (10, 28, 29). Perturbation of the mannan outer layer leads to β-glucan exposure and enhanced immune recognition (10, 30, 31). This model is consistent with immune evasion strategies in other pathogens. For example, the Cryptococcus neoformans cell wall is coated with a glucuronoxylomannan polysaccharide capsule that protects cells from phagocytosis (32). Histoplasma capsulatum chemotype II strains mask β-glucan from immune recognition under an outer layer of α-glucan (33). However, simply masking β-glucan by a cell wall outer layer is not sufficient to account for the phenomenon of lactate-induced β-glucan masking in C. albicans because a range of mannan mutants still display reductions in β-glucan exposure in response to lactate (15). Even in wild-type cells, clearly some glucan escapes mannan-mediated masking to generate foci of exposure at the C. albicans cell surface (e.g., Fig. 4A).

**FIG 8:**
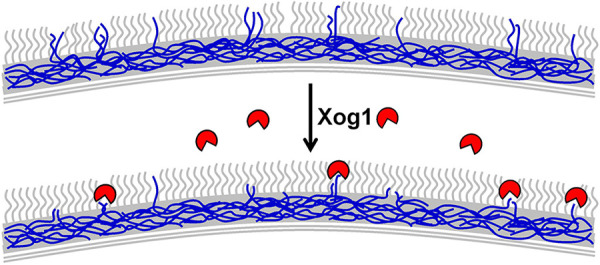
Xog1 “shaves” β-1,3-glucan to facilitate immune evasion by C. albicans. Top panel: patches of β-1,3-glucan (blue) are accessible to dectin-1 recognition in glucose-grown C. albicans cells. Bottom panel: specific host inputs induce Xog1 secretion. Xog1 exoglucanase activity (red) shaves exposed β-1,3-glucan, thereby reducing its accessibility to dectin-1 recognition.

We show that C. albicans expresses a major extracellular exoglucanase, Xog1, in response to lactate. Xog1 expression both is regulated by the same pathways that control lactate-induced β-glucan masking and is required for this phenotype. Other activities may contribute to lactate-induced reductions in β-glucan exposure (Fig. 3A), but Xog1 is the major extracellular exoglucanase under these experimental conditions. Significantly, Xog1 inactivation attenuates immune evasion by preventing the decrease in phagocytosis rates and the tempering of cytokine elicitation normally associated with lactate responses in C. albicans. These effects were phenocopied by the pharmacological inhibition of exoglucanase by castanospermine, reinforcing the idea that the Xog1 exoglucanase is a major factor in β-glucan masking and manipulates host immune recognition. The induction of Xog1 by C. albicans in response to lactate is particularly significant, because this suggests that exoglucanase production is an anticipatory response that protects the fungus against impending attack by innate immune cells (34). Given the biochemical role of Xog1, we suggest that this exoglucanase “shaves” β-glucan epitopes from the C. albicans cell surface, rather than physically masks them (Fig. 8).

However, *XOG1* inactivation does not significantly attenuate virulence in G. mellonella (see Fig. S2 in the supplemental material) or significantly reduce kidney burdens in mice (22). Interestingly, H. capsulatum expresses an endoglucanase, Eng1, which, together with the outer α-1,3-glucan layer, reduces β-1,3-glucan exposure and promotes immune evasion by H. capsulatum cells (35). Blocking *ENG1* expression in H. capsulatum only leads to a slight reduction in fungal burden in mice after 8 days of infection (35).

Clearly, Xog1 plays a major role in lactate-induced β-1,3-glucan shaving. Nevertheless, we note that our proteomics data sets reveal other glucanases that were differentially regulated in response to lactate. These enzymes could potentially contribute to PAMP remodeling. Also, we cannot exclude minor roles for other glucanases *in vivo* and, in particular, the putative exoglucanase, Exg2, which is transcriptionally induced in response to lactate. but was not detected in our proteomics data sets (15), and which might be secreted at elevated levels *in vivo*. It is also conceivable that environmentally contingent changes in the outer mannan layer might affect the efficacy with which this layer masks β-glucan. Indeed, the abundance of some cell wall mannoproteins did change in response to lactate (Table S2). Therefore, while Xog1 plays a major role in β-glucan shaving *in vitro*, other mechanisms could potentially contribute to the observed changes in β-glucan exposure that occur during the adaptation of C. albicans to certain specific host signals (15–18, 36). Furthermore, we cannot exclude the possibility that the inactivation of Xog1 influences innate immune responses in some other way, for example by indirectly influencing the levels of certain cell wall proteins, or enhancing PAMP recognition by some means.

10.1128/mBio.00984-20.4 TABLE S2 Relative quantification of secreted and cell wall proteins from C. albicans during lactate- and hypoxia-induced masking. Download Table S2, PDF file, 0.3 MB. Copyright © 2020 Childers et al. 2020 Childers et al. This content is distributed under the terms of the Creative Commons Attribution 4.0 International license.

Is exoglucanase a viable antifungal drug target? Our results show that the inhibition of exoglucanase with the small molecule castanospermine enhanced immune recognition of C. albicans cells and significantly improved the survival of G. mellonella larvae after lethal challenge with the fungus. Although castanospermine does not inhibit endoglucanase *in vitro* (24), potential effects upon activities other than exoglucanase cannot be excluded *in vivo.* For example, host cells also produce glucanases and α-glucosidases that can be inhibited by castanospermine, leading to mammalian toxicity (26). Less toxic derivatives of castanospermine are under development for the treatment of viral infections, including dengue fever (37). These drugs are thought to inhibit viral protein folding by blocking the trimming of *N*-linked carbohydrates (38). Taken together, the data suggest that the development of fungus-specific exoglucanase inhibitors might provide a new therapeutic avenue to augment current antifungal treatments.

## MATERIALS AND METHODS

### Strains and growth conditions.

Strains used in this study are listed in Table S1 in the supplemental material. Strains were cultured routinely on YPD agar (1% yeast extract, 2% Bacto peptone, 2% glucose, and 2% Bacto agar) at 30°C. For β-glucan masking experiments, strains were grown at 30°C, 200 rpm, overnight in yeast nitrogen base without amino acids (BD Difco; 6.7 g/liter) containing the appropriate supplements, plus 2% glucose (YNB-Glu) and then diluted to an OD_600_ of ∼0.1 in fresh YNB-Glu with and without 2% d/l-lactate (Sigma) and grown 5 h at 30°C, 200 rpm. To prepare hypoxic cultures, cells were inoculated into YNB-Glu in a screw-cap conical flask under nitrogen (16). Cells treated with castanospermine (Cayman Chemical) were grown as described above for β-glucan masking experiments with the addition of castanospermine (in dimethyl sulfoxide [DMSO]) to a final concentration of 1 μM.

### Strain construction.

Heterozygous (*XOG1/xog1*Δ) and homozygous (*xog1*Δ*/xog1*Δ) C. albicans mutants were constructed sequentially using the *SAT1* flipper (39). Briefly, upstream and downstream flanking regions to the *XOG1* open reading frame were amplified using primers 1 to 4 listed in Table S1 and cloned into the *SAT1* flipper plasmid between the NotI/SacII and KpnI/XhoI restriction sites, respectively. The resultant plasmid was digested with KpnI/SacII to release the disruption cassette, which was then purified by gel extraction (Qiagen) and transformed into C. albicans SC5314 using a lithium acetate protocol (40). C. albicans reintegrant strains (*xog1*Δ*/xog1*Δ*/XOG1*) were constructed by amplifying 2 kb of *XOG1* promoter region, the open reading frame, and terminator from C. albicans SC5314 genomic DNA and cloning the PCR product into plasmid CIp-NAT at the NotI/SacII restriction sites (41). The resulting plasmid was linearized with StuI and transformed into the *xog1*Δ*/xog1*Δ mutant for integration at the *RPS1* locus. Mutant genotypes were confirmed by colony PCR.

### Proteomics.

C. albicans wild-type (SC5314), *crz1*Δ, and *gpr1*Δ *gpa2*Δ strains were grown for 5 h in YNB-Glu, YNB-Glu plus Lac, or YNB-Glu under hypoxic conditions to induce β-glucan masking. Cell wall extracts were prepared from cells by an SDS boiling procedure (42). Briefly, cells were washed twice with 4°C distilled water (dH_2_O) and broken with 400- to 600-nm acid-washed glass beads using a FastPrep machine (MP Biomedical). Cell homogenates were pelleted and washed in 1 M NaCl and resuspended in SDS extraction buffer (50 mM Tris-HCl, pH 7.5, 2% SDS, 0.3 M β-mercaptoethanol, and 1 mM EDTA). Homogenates were boiled at 100°C for 10 min, washed with dH_2_O, and freeze-dried. Cell wall protein extracts (lyophilized, 1 mg) or secreted proteins (approximately 10 μg) were resuspended in 100 μl ammonium bicarbonate (50 mM) in 1.5-ml low-binding tubes. Reduction was performed by the addition of 2 μl dithiothreitol (DTT; 200 mM) with incubation for 30 min at 60°C and then alkylation by the addition of 4 μl iodoacetamide (200 mM) with incubation in the dark for 30 min at 25°C. Proteins were digested by the addition of 10 μl sequencing-grade trypsin (20 μg/ml, Promega) with incubation overnight at 37°C. Digested cell wall samples were centrifuged at 17,000 × *g*, and the supernatants were transferred to clean tubes. The pellets were resuspended in 50 μl 0.1% (vol/vol) trifluoroacetic acid (TFA)-70% (vol/vol) acetonitrile in ultrapure water, shaken for 10 min, and centrifuged again, and the supernatants were added to those already taken. All digests were frozen at −70°C and dried by vacuum centrifugation (SpeedVac SC110A; Savant). Peptides were dissolved in 40 μl 0.1% TFA, desalted on μ-C_18_ ZipTips (Merck Millipore, Watford, United Kingdom), and dried by vacuum centrifugation.

Desalted peptide samples were dissolved in 10 μl loading solvent (0.1% [vol/vol] formic acid, 2% [vol/vol] acetonitrile in ultrapure water). Liquid chromatography-tandem mass spectrometry was performed using a Q Exactive Plus/Ultimate 3000 RSLCnano system (Thermo Scientific, Hemel Hempstead, United Kingdom). Sample (5 μl) was loaded onto a trapping column (C_18_ PepMap 100; 300-μm inside diameter [i.d.] by 5 mm) for 5 min at 10 μl/min and reverse-flushed to the nanocolumn (PepMap RSLC C_18_; 75-μm i.d. by 25 cm) at 300 μl/min using a gradient of various proportions of solvent A (0.1% formic acid in ultrapure water) and solvent B (0.1% formic acid, 80% acetonitrile in ultrapure water). The proportion of solvent B was increased from 3 to 10% during 5 to 10 min, from 10 to 40% during 10 to 40 min, and from 40 to 80% during 40 to 45 min; held at 80% during 45 to 53 min; and then returned to 3%. Mass spectra were acquired during 5 to 65 min in full MS/data-dependent MS2 mode using a “Top 10” method. MS1 scans were performed from 375 to 1,750 *m/z* with a resolution of 70,000, an automatic gain control (AGC) target of 3e6, and maximum injection time (max IT) of 50 ms. The 10 most abundant precursor ions with charge states of +2 to +5 were selected from each MS1 scan for sequential trapping and fragmentation by higher-energy collisional dissociation (normalized collision energy 26%). MS2 scans were performed with a resolution of 17,500, an AGC target of 5e4, and max IT of 100 ms. Previously selected ions were dynamically excluded for 40 s, and peptide ions were preferred.

Raw data files from the Q Exactive Plus were processed using Proteome Discoverer v1.4 (Thermo Scientific) with Mascot v2.5 (Matrix Science, London, United Kingdom) as the search engine. The database comprised 12,421 protein sequences in the file <C_albicans_SC5314_A22_current_orf_trans_all.fasta> downloaded from the Candida Genome Database (www.candidagenome.org, datestamp 2016-02-19). Search parameters were: enzyme = trypsin; maximum missed cleavage sites = 2; instrument = electrospray ionization Fourier transform ion cyclotron resonance (ESI-FTICR); precursor mass tolerance = 10 ppm; fragment mass tolerance = 20 millimass units (mmu); dynamic modifications = oxidation (M); static modifications = carbamidomethyl (C). Results were filtered for peptides with a Mascot significance value of 0.05, and protein area values were based on the extracted ion chromatograms of the three most abundant peptides. Peptide-spectrum matches were validated using a decoy database search with target false-discovery rates of 0.01 (strict) and 0.05 (relaxed). To perform a semiquantitative analysis, the number of detected peptides for each protein was divided by the total number of peptides detected for the respective biological replicate and multiplied by 100 to obtain the percent spectral count. Percent spectral counts were averaged between biological replicates to calculate percent spectral means for each protein.

### Flow cytometry.

Cells were inactivated overnight at room temperature in 50 mM thimerosal, washed three times and resuspended in phosphate-buffered saline (PBS), and counted by hemocytometer (15). Then, 2.5 × 10^6^ cells were washed in fluorescence-activated cell sorting (FACS) buffer (PBS containing 1% fetal bovine serum [FBS], 0.5 mM EDTA) and stained with Fc-dectin-1 and goat (Gt) F(ab′)_2_ anti-human IgG conjugated to Alexa Fluor 488 (Invitrogen), and 10,000 cells from each experiment were analyzed by either FACSCalibur or BD Fortessa. The data represent three biological replicates for each condition.

### Microscopy.

For microscopy, cells were inactivated and stained as described above for flow cytometry with the addition of concanavalin A conjugated to Texas Red (Invitrogen) to stain cell wall mannan. Cells were imaged using phase-contrast and fluorescence microscopy on a Zeiss Axioplan 2 microscope. Images were captured with a Hamamatsu C4742-95 digital camera (Hamamatsu Photonics) and recorded using Zeiss Zen software (Oberkochen, Germany).

### Exoglucanase assay.

Exoglucanase activity was determined by a para-nitrophenol-β-d-glucopyranoside (PNPG; Sigma) cleavage assay (23). Cell supernatants (10 ml) were filtered and concentrated to 1 ml on 10-kDa-cutoff Vivaspin protein concentrator columns (GE Healthcare and Life Sciences) and washed in 50 mM sodium acetate, pH 5.3. One hundred twenty-five microliters of concentrated supernatant was coincubated with an equal volume of PNPG (5 mg/ml in 50 mM sodium acetate, pH 5.3) for 16 h at 37°C. Reactions were stopped with 2.25 ml of Na_2_CO_3_ (40 g/liter), the absorbance was measured at 410 nm, and values were normalized to cell density. Assays were performed in duplicate, and blanks (250 μl 50 mM sodium acetate, pH 5.3) and negative controls (equal volumes 50 mM sodium acetate, pH 5.3, and PNPG) were included. Relative exoglucanase activities were calculated as follows: (normalized *A*_410 Glu+Lac_/normalized *A*_410 Glu_) × 100. Strains were assayed in biological triplicate.

### Supernatant complementation.

C. albicans wild-type (SC5314) and *czf1Δ* cells were grown overnight in 5 ml YNB-Glu at 30°C, 200 rpm. The wild-type cells were diluted to an OD_600_ of ∼0.1 into 50 ml fresh YNB-Glu with or without 2% d/l-lactate and cultured at 30°C, 200 rpm, for 4 h, and at this point, cells in one portion of the culture were inactivated in 50 mM thimerosal for analysis. The supernatant from the other portion of the culture was passed through a 0.45-μm syringe filter: one-half was used directly, and the other half was boiled at 100°C for 15 min and cooled on ice. The C. albicans *czf1Δ* cells were diluted to an OD_600_ of ∼0.1 in this spent-boiled medium, in the spent-untreated medium, or in fresh medium that had been prewarmed to 30°C and were incubated for 4 h at 30°C, 200 rpm. Cells were then harvested and inactivated in 50 mM thimerosal for flow cytometry (above).

### Macrophage interactions.

Bone marrow-derived macrophages (BMDMs) were prepared from the femurs and tibias of 12-week-old male C57BL/6 mice. BMDMs were differentiated for 7 days in Dulbecco’s modified Eagle’s medium (DMEM; Gibco) containing 10% heat-inactivated fetal calf serum supplemented with 15% L-cell conditioned medium (43). C. albicans cells grown in glucose plus lactate or glucose alone were inactivated with thimerosal, mixed with the BMDMs at a ratio of 3:1 (yeast cells to macrophages), and imaged at 2-min intervals for 2 h using a Nikon Eclipse Ti UltraVIEW VoX spinning disk microscope with Volocity software (Quorum Technologies, ON, Canada). The number of C. albicans cells engulfed per macrophage was quantified over 2 h. The difference in engulfment between conditions was determined in GraphPad Prism 8 using one-way ANOVA with Tukey’s *post hoc* test.

### Cytokine measurements.

PBMCs were prepared from nonheparinized whole blood (40 ml) collected from three healthy volunteers by Ficoll-Paque centrifugation according to the manufacturer’s instructions (Sigma-Aldrich). Thimerosal-fixed C. albicans cells were washed thrice with sterile PBS and incubated for 24 h with PBMCs (5:1, yeast to PBMCs). The coincubation supernatant was collected, and specific cytokines were quantified with a Luminex screening kit (R&D Systems) in the Bioplex 200 system (Bio-Rad) according to the manufacturer’s instructions.

### Galleria mellonella infections.

*Galleria* larvae were acquired from BioSystems Technology and stored with wood shavings in the dark at 20°C prior to the experiment. C. albicans wild-type (SC5314) cells were grown overnight, diluted into fresh glucose medium, and grown for a further 5 h to reach exponential phase. Cells were collected by centrifugation, washed thrice in PBS, and resuspended in sterile PBS. Groups of 10 G. mellonella larvae (∼300-mg weight) were inoculated with a 50-μl suspension of 5 × 10^5^ C. albicans yeast cells in the last left proleg (27). Larvae received a second 50-μl injection of either 1 μM castanospermine or vehicle in the last right proleg. Injections were delivered with U-100 30G BD Micro-fine syringes (BD). Control groups of five larvae received PBS alone or PBS and castanospermine injections in 50-μl suspensions. The larvae were then incubated in the dark at 37°C, and survival was assessed every 2 to 4 h thereafter. No larvae in the control group died over the course of the experiment. Survival experiments were terminated approximately 5 days postinfection. The virulence of *xog1Δ* cells was assessed as described above, with the following modification: G. mellonella larvae were inoculated with a 50-μl suspension of 1 × 10^5^ C. albicans wild-type (SC5314) or *xog1Δ* yeast cells in the last left proleg.

### Statistics.

G. mellonella survival was assessed by Mantel-Cox test using SPSS software. All other statistical analyses were performed using GraphPad Prism 8 software.

### Ethics.

This study complies with all relevant ethical regulations for experiments with human participant samples. Donor blood was collected from healthy volunteers with their informed consent according to local guidelines and regulations that were approved by the College Ethics Review Board of the University of Aberdeen (CERB/2016/8/1300). Animal usage was approved by the University of Aberdeen Animal Welfare and Ethical Review body. Mice were bred in-house, housed in stock cages under specific-pathogen-free conditions, and selected at random. Animals did not undergo any regulated procedures prior to culling by cervical dislocation.

### Data availability.

The data supporting the findings in this study are available within the paper and accompanying supplemental material. The mass spectrometry proteomics data have been deposited to the ProteomeXchange Consortium via the PRIDE (44) partner repository with the data set identifiers PXD018027 and PXD018044.

10.1128/mBio.00984-20.10 MOVIE S6 Second time-lapse video of BMDM interactions with C. albicans cells grown on glucose plus lactate in the presence of castanospermine. This movie shows the first 2 h of interactions between murine BMDMs and wild-type C. albicans SC5314 cells. It is representative of 12 movies in total (4 movies from 3 mice). Download Movie S6, AVI file, 18.4 MB. Copyright © 2020 Childers et al. 2020 Childers et al. This content is distributed under the terms of the Creative Commons Attribution 4.0 International license.
