# Supplementary material for: Epitope Shaving Promotes Fungal Immune Evasion
Source: mBio. 2020 Jul 7;11(4):e00984-20. doi: 10.1128/mBio.00984-20 (PMC7343991; doi:10.1128/mBio.00984-20)
Supplement: FIG S1 [file mBio.00984-20-sf001.pdf]

Figure S1

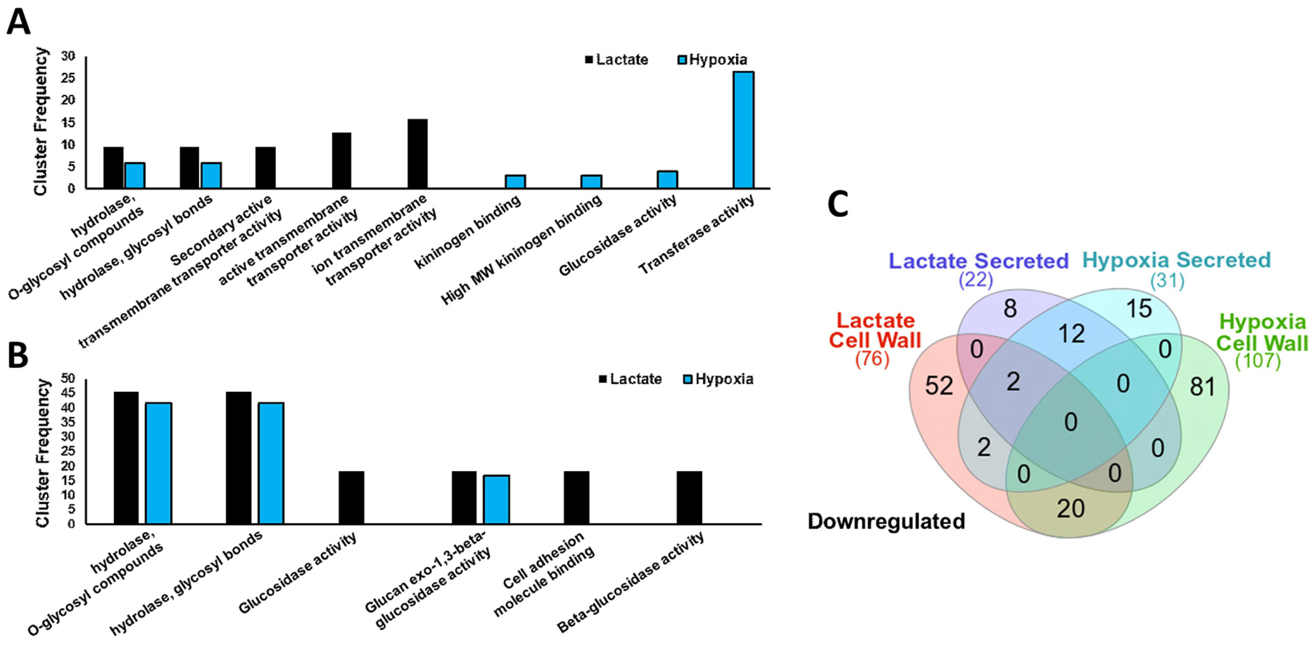

Figure S1: Hydrolase and glucosidase activities are represented at higher frequency in β-glucan masking cells compared to glucose-grown cells. GO Molecular Function analysis was performed for upregulated peptides in β-glucan masking conditions compared to glucose-grown controls from (A) cell wall proteomics and (B) secretomics. (C) Venn diagram of proteins with <0.5 fold change in peptide spectral mean (PSM) from cells grown in lactate or hypoxia compared to glucose. n=4
