# Supplementary material for: Epitope Shaving Promotes Fungal Immune Evasion
Source: mBio. 2020 Jul 7;11(4):e00984-20. doi: 10.1128/mBio.00984-20 (PMC7343991; doi:10.1128/mBio.00984-20)
Supplement: TABLE S1 [file mBio.00984-20-st001.pdf]

Table S1. Strains and oligos used in this study

Strain	Genotype	Parent	Reference
SC5314			(Gillum et al. 1984)
czf1Δ	*czf1Δ::hisG/czf1Δ::hisG*	CAI4	(Brown et al. 1999)
crz1Δ	*crz1Δ::hisG/crz1Δ::hisG::URA3::hisG*	CAF4-2	(Karababa et al. 2006)
gpr1Δgpa2Δ	*gpa2Δ::hisG/gpa2Δ::hisG gpr1Δ::hisG/gpr1Δ::hisG-URA3-hisG*	CAI4	(Maidan et al. 2005)
xog1Δ/+	*XOG1/xog1Δ::frt*	SC5314	This study
xog1Δ/Δ	*xog1Δ::frt/xog1Δ::frt*	*xog1Δ/+*	This study
xog1Δ/XOG1	*xog1Δ::frt/xog1Δ::frt RPS1/RPS1::XOG1-NAT1*	*xog1Δ/Δ*	This study

Oligo	Sequence [a]	Description
DCO58	agtactgaaggtaccGAGTTCAACCAGCTTTTC	5' *XOG1* primer for 5' *XOG1* 'outer' flank with *KpnI* site for cloning into *SAT* flipper
DCO59	agtactgaactcgagGAAATTCAGAAATTCAGG	3' *XOG1* primer for 5' *XOG1* 'outer' flank with *XhoI* site for cloning into *SAT* flipper
DCO54	agtactgaaggtaccCCAAAAGTTTCATTATTCC	5' *XOG1* primer for 5' *XOG1* 'inner' flank with *KpnI* site for cloning into *SAT* flipper
DCO55	agtactgaactcgagAGCTGATTATGTATTAATC	3' *XOG1* primer for 5' *XOG1* 'inner' flank with *XhoI* site for cloning into *SAT* flipper
DCO56	agtactgaagcggccgcCGTCTTGATTCTACAAGAGC	5' *XOG1* primer for 3' *XOG1* flank with *NotI* site for cloning into *SAT* flipper
DCO57	agtactgaaccgcggGAAAAAATACAACAAAGCG	3' *XOG1* primer for 3' *XOG1* flank with *SacII* site for cloning into *SAT* flipper
DCO53	agtactgaaccgcggCAAACCAGTAGATGACAGC	5' *XOG1* primer for cloning *XOG1*, 2 kb promoter, and 500 bp terminator into *CIp-NAT* with *SacII* site
DCO49	agtactgaagcggccgcGTATCTAAAACTAACGTGG	3' *XOG1* primer for cloning *XOG1*, 2 kb promoter, and 500 bp terminator into *CIp-NAT* with *SacII* site
DCO39	GAAAGACCAAGTCGCTTGC	5' *XOG1 SAT* flipper integration check
DCO64	CGACTACATCAATGAAATCC	3' *SAT* flipper integration check
DCO43	CTTTTGAATACACTGGAGGC	5' *XOG1* ORF check
DCO44	AGTGAATACTTTTTTGCC	3' *XOG1* ORF check

a Lowercase bases indicate flanking sequences within primers that contain restriction sites for cloning.
