# Supplementary material for: Epitope Shaving Promotes Fungal Immune Evasion
Source: mBio. 2020 Jul 7;11(4):e00984-20. doi: 10.1128/mBio.00984-20 (PMC7343991; doi:10.1128/mBio.00984-20)
Supplement: FIG S2 [file mBio.00984-20-sf002.pdf]

Figure S2

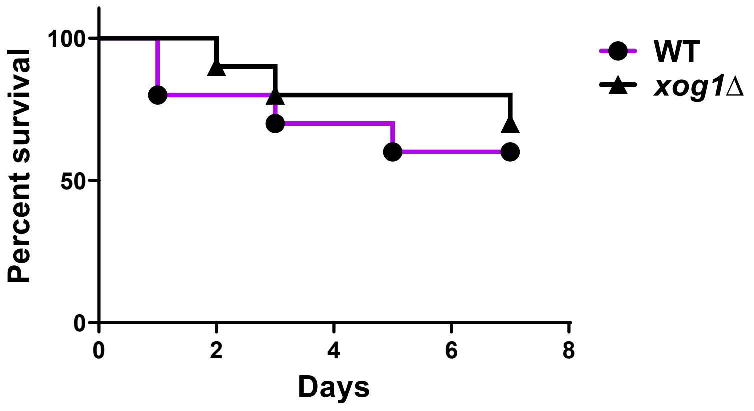

Figure S2: *XOG1* inactivation does not significantly attenuate *C. albicans* virulence. Percentage survival of *G. mellonella* larvae infected with wild type or *xog1Δ C. albicans* cells (n=10).
