# Supplementary material for: Epitope Shaving Promotes Fungal Immune Evasion
Source: mBio. 2020 Jul 7;11(4):e00984-20. doi: 10.1128/mBio.00984-20 (PMC7343991; doi:10.1128/mBio.00984-20)
Supplement: TABLE S2 [file mBio.00984-20-st002.pdf]

Table S2. Relative quantification of secreted and cell wall proteins from *C. albicans* during lactate- and hypoxia-induced masking.

	Protein	Alias	Average Glu PSM (n=4)	Average GluLac PSM (n=4)	Fold Change GluLac/Glu PSM
**WILD TYPE SECRETOME: Glucose + Lactate *versus* Glucose**					
**Upregulated**	XOG1		0.45	5.61	12.45
	C1_02990C_B	XOG1	0.23	5.38	23.88
	CHT3		1.65	3.43	2.07
	ATC1		1.02	1.83	1.80
	SCW11		0.73	1.77	2.41
	PGA4		1.32	2.40	1.82
	ALS2		0.45	0.67	1.49
	ENG1		0.45	1.68	3.73
	ALS3		0.40	1.07	2.65
	TAL1		0.28	0.58	2.04
	CRH11		n.d.	0.64	>0.64
	ALS4		n.d.	0.27	>0.27
**Downregulated**	DAG7		2.37	1.07	0.45
	NUP		2.06	0.80	0.39
	ATP1		2.23	0.80	0.36
	RPL30		1.32	0.14	0.11
	RPL14		1.32	0.53	0.40
	IPP1		0.69	0.23	0.33
	CIT1		1.10	0.30	0.27
	FBA1		1.32	0.14	0.11
	EFT2		1.32	0.43	0.32
	RPL25		0.69	n.d.	0.00
	C2_06550W_A		0.41	0.13	0.32
	SOD4		1.32	0.13	0.10
	GLR1		0.91	0.27	0.30
	CDC19		0.40	n.d.	0.00
	C3_04810C_B		1.10	0.14	0.13
	HSP60		0.28	n.d.	0.00
	EFB1		0.69	0.27	0.39
	RPL11		0.28	n.d.	0.00
	RPL13		0.92	n.d.	0.00
	RPL35		0.23	n.d.	0.00
	RPS42		0.23	n.d.	0.00
	TPI1		0.41	n.d.	0.00

	Protein	Alias	Average Glu PSM (n=4)	Average Hypoxia PSM (n=4)	Fold Change Hypoxia/Glu PSM
**WILD TYPE SECRETOME: Hypoxia *versus Normoxia***					
**Upregulated**	SIM1		2.24	3.84	1.71
	XOG1		0.45	3.13	6.95
	C1_02990C_B	XOG1	0.23	2.76	12.25
	ATC1		1.02	1.71	1.68
	SCW11		0.73	2.25	3.06
	UTR2		0.73	1.87	2.54
	C1_07030C_B	RBT4	0.92	2.09	2.27
	RBT4		0.92	2.25	2.45
	ENG1		0.45	1.17	2.61
	C2_05410W_B		0.51	0.80	1.58
	TAL1		0.28	0.43	1.53
	YWP1		0.23	0.53	2.36
	CRH11		n.d.	0.37	3.69
	ALS4		n.d.	0.80	8.03
**Downregulated**	ENO1		7.82	3.10	0.40
	NUP		2.06	0.64	0.31
	HSP70		1.32	0.16	0.12
	ATP1		2.23	n.d.	0.00
	ALS2		0.45	0.16	0.37
	RPL30		1.32	0.64	0.48
	RPS7A		1.32	0.16	0.12
	ASN1		1.32	0.64	0.48
	FBA1		1.32	0.16	0.12
	MSB2		1.32	0.60	0.45
	COI1		0.92	0.20	0.22
	GPM1		0.51	0.16	0.32
	ASC1		1.32	0.16	0.12
	RPL5		1.04	0.27	0.26
	RPL25		0.69	0.27	0.39
	FAS2		0.69	n.d.	0.00
	GUA1		1.04	n.d.	0.00
	SAP10		0.23	n.d.	0.00
	SOD4		1.32	0.20	0.15
	GLR1		0.91	0.16	0.18
	YST1		0.28	n.d.	0.00
	C3_04810C_B		1.10	n.d.	0.00
	HSP60		0.28	n.d.	0.00
	EFB1		0.69	n.d.	0.00
	C3_02880W_B		0.63	n.d.	0.00
	RPL13		0.92	n.d.	0.00
	ATP2		0.28	n.d.	0.00

	TEF2		0.23	n.d.	0.00
	RPL35		0.23	n.d.	0.00
	TPI1		0.41	n.d.	0.00

WILD TYPE CELL WALL PROTEOME: Glucose + Lactate *versus* Glucose

	Protein	Alias	Average Glu PSM (n=4)	Average GluLac PSM (n=4)	Fold Change GluLac/Glu PSM
**Upregulated**	CHT2		0.85	1.71	2.00
	RHD3		1.03	2.13	2.06
	TEF2		1.27	2.50	1.96
	ECM33		0.74	1.24	1.68
	MP65		0.82	1.34	1.63
	EFT2		1.27	1.92	1.51
	PET9		1.03	1.64	1.59
	ALS1		0.48	0.81	1.67
	ALS4		0.32	0.56	1.75
	TDH3		0.37	0.69	1.85
	CRH11		0.40	0.80	2.00
	C4_00130W_B		0.23	0.38	1.65
	PGK1		0.03	0.15	5.28
	UBI3		0.35	0.58	1.65
	PGA10		0.12	0.19	1.56
	C7_00860W_B		0.18	0.28	1.57
	C1_13860C_B		0.16	0.27	1.62
	CR_04100C_B		0.26	0.41	1.59
	C3_01550C_B		0.25	0.40	1.58
	PGA4		0.36	0.78	2.16
	C5_01540W_A		0.46	0.76	1.64
	ACO1		0.41	0.77	1.87
	SIM1		0.26	0.53	2.01
	YWP1		0.22	0.42	1.92
	RPL4B		0.21	0.51	2.50
	C6_03760C_B		0.43	0.64	1.49
	C1_03090W_B		0.23	0.58	2.49
	RPS3		0.09	0.23	2.72
	PNG2		0.04	0.08	1.92
	MIR1		0.23	0.63	2.71
	RPL19A		0.16	0.52	3.16
	CDC60		0.23	0.46	2.03
	C2_06810C_B		0.08	0.29	3.48
	C2_08160C_B		0.05	0.11	2.16
	UTR2		0.08	0.29	3.76
	C1_01480C_B		0.20	0.49	2.53
	RPS6A		0.13	0.28	2.20
	RPL6		0.05	0.10	1.97
	POR1		0.05	0.26	4.74
	TIF		0.08	0.46	5.70
	C4_04890C_B		0.08	0.46	5.93
	ILV2		0.07	0.25	3.44
	SEO1		0.21	0.33	1.57
	ACT1		0.03	0.15	4.71
	C2_08870C_B		0.12	0.23	1.91
	DBP3		0.17	0.34	1.98
	TOM70		0.15	0.21	1.45
	C1_06450C_B		0.09	0.35	3.72
	C1_03020C_B		0.05	0.16	2.90
	RPS5		0.10	0.24	2.43
	RPL12		0.04	0.15	3.71
	RPL2		0.08	0.16	2.10
	C3_05100C_B		0.05	0.13	2.59
	RPS26A		0.05	0.16	2.92
	LAT1		0.13	0.22	1.76
	C1_01640W_B		0.02	0.11	5.87
	RPS13		0.07	0.32	4.42
	TSA1		0.02	0.06	3.60
	C1_05720W_A		0.02	0.03	1.74
	SDH12		0.10	0.16	1.61
	BGL2		0.12	0.19	1.53
	RPL43A		0.05	0.19	3.54
	C3_04910C_B		0.03	0.06	1.72
	C1_06580W_B		0.04	0.21	5.77
	COX5		0.01	0.08	5.46
	C2_03810C_B		0.08	0.31	4.00
	C2_04460W_B		0.02	0.08	4.29
	COX9		0.08	0.29	3.72
	CR_07630C_B		0.04	0.16	4.00
	C1_10870W_B		0.04	0.07	1.66
	GUS1		0.10	0.18	1.79
	C7_00960W_B		0.04	0.10	2.62
	C1_08110W_A		0.11	0.19	1.77
	C1_00420W_A		0.04	0.06	1.76
	C1_02460W_B		0.10	0.26	2.57
	ENG1		0.07	0.17	2.43
	TUP1		0.01	0.03	2.37
	C1_11670W_B		0.03	0.09	2.70
	CAM1		0.01	0.04	3.09
	C2_05500W_B		0.01	0.07	4.63

	SAM2	0.03	0.05	1.66
	C1_01530C_A	0.03	0.07	2.04
	SHM1	0.04	0.07	1.86
	ILV3	0.02	0.05	2.96
	C2_03820C_B	0.08	0.19	2.42
	MTS1	0.02	0.07	4.17
	C2_04950C_B	0.02	0.03	1.86
	C7_00990W_B	0.02	0.11	5.87
	LAG1	0.03	0.09	2.70
	RPO41	0.07	0.12	1.67
	CDR4	0.01	0.02	1.54
	C2_05410W_B	0.04	0.21	5.32
	ZUO1	0.01	0.05	3.76
	RPL10A	0.04	0.13	3.17
	PDI1	0.01	0.02	1.54
	ADE12	0.02	0.03	1.74
	CHS3	0.01	0.02	1.54
	YHM2	0.01	0.11	7.47
	C3_05280C_A	0.01	0.02	1.54
	TNA1	0.03	0.04	1.54
	C6_03790C_B	0.01	0.02	1.54
	UTP21	0.04	0.06	1.56
	C7_03050W_B	0.04	0.11	2.62
**Downregulated**	C4_06570C_B	0.34	0.14	0.41
	ENO1	0.12	0.05	0.45
	PFK1	0.43	0.13	0.30
	C4_00890W_B	0.83	0.32	0.39
	ARO1	0.82	0.30	0.37
	NIP1	0.70	0.27	0.38
	PGI1	0.08	0.00	0.00
	SPF1	0.58	0.21	0.37
	C5_02170C_B	0.33	0.13	0.39
	GPH1	0.17	0.00	0.00
	ELF1	0.52	0.15	0.30
	C1_04970W_A	0.46	0.22	0.47
	CPA2	0.49	0.20	0.42
	FBA1	0.10	0.00	0.00
	C2_06640C_B	0.41	0.21	0.50
	GPM1	0.10	0.00	0.00
	TKL1	0.30	0.09	0.29
	MLT1	0.45	0.15	0.34
	ACS2	0.31	0.09	0.28
	ILV5	0.02	0.00	0.00
	PGA63	0.16	0.05	0.32
	C1_08090C_B	0.51	0.14	0.28
	CDC48	0.27	0.11	0.41
	UGP1	0.11	0.02	0.19
	C2_02540W_A	0.22	0.11	0.49
	MET15	0.13	0.02	0.17
	OLE1	0.27	0.12	0.44
	PHM7	0.29	0.14	0.49
	HSP104	0.12	0.00	0.00
	NDE1	0.27	0.13	0.48
	MET3	0.09	0.02	0.25
	C1_03370W_A	0.24	0.12	0.49
	ADE17	0.18	0.02	0.12
	CR_07400C_B	0.14	0.06	0.45
	C7_00570W_B	0.28	0.09	0.31
	C1_01590C_A	0.21	0.09	0.41
	C1_13780W_B	0.28	0.09	0.31
	TPI1	0.10	0.00	0.00
	CR_10140W_A	0.19	0.04	0.23
	CR_04510W_B	0.10	0.00	0.00
	CR_04380C_B	0.15	0.02	0.14
	CR_10100C_B	0.12	0.00	0.00
	SUR7	0.07	0.02	0.29
	RPL8B	0.03	0.00	0.00
	C7_03670W_B	0.22	0.06	0.25
	OPT3	0.01	0.00	0.00
	C7_03660C_A	0.16	0.02	0.13
	MES1	0.27	0.05	0.20
	FET99	0.22	0.11	0.48
	C6_02260C_B	0.06	0.03	0.49
	C5_04700C_B	0.27	0.05	0.20
	CAM1-1	0.09	0.02	0.25
	DCK1	0.13	0.04	0.34
	C7_04300W_B	0.18	0.03	0.18
	C4_06110C_B	0.18	0.00	0.00
	ZWF1	0.10	0.00	0.00
	PRT1	0.11	0.03	0.27
	IST2	0.11	0.03	0.32
	C1_14080W_A	0.15	0.03	0.21
	RPG1A	0.22	0.11	0.47
	HHF1	0.04	0.00	0.00
	C1_11660W_B	0.03	0.00	0.00
	UBA1	0.07	0.00	0.00
	C3_04820C_B	0.11	0.03	0.30

	CR_02860W_B		0.05	0.02	0.40
	AQY1		0.04	0.00	0.00
	SEC24		0.08	0.00	0.00
	CR_05520W_B		0.16	0.02	0.14
	SNQ2		0.06	0.00	0.00
	COX2		0.06	0.02	0.38
	C2_03080W_B		0.04	0.00	0.00
	C4_04160W_B		0.04	0.00	0.00
	C6_00440C_B		0.05	0.00	0.00
	ACH1		0.05	0.02	0.47
	C4_05240C_B		0.07	0.00	0.00
	C4_03390W_B		0.05	0.00	0.00
	YCF1		0.11	0.02	0.19
	NAD5		0.07	0.02	0.32
	C1_03380W_B		0.02	0.00	0.00
	MLP1		0.07	0.00	0.00
	C4_05330C_A		0.05	0.00	0.00
	C4_04410C_B		0.10	0.02	0.22

WILD TYPE CELL WALL PROTEOME: Hypoxia *versus Normoxia*

	Protein	Alias	Average Glu PSM (n=4)	Average Hypoxia PSM (n=4)	Fold Change Hypoxia/Glu PSM
**Upregulated**	TDH3		0.37	1.23	3.30
	C4_06570C_B		0.34	1.01	2.92
	CRH11		0.40	0.64	1.59
	GDH3		0.41	0.96	2.33
	ENO1		0.12	0.91	7.66
	PGK1		0.03	0.97	34.30
	HGT7		0.38	0.58	1.52
	PFK1		0.43	0.98	2.29
	C1_13860C_B		0.16	0.46	2.76
	C2_05460W_B		0.20	0.82	4.16
	CR_04100C_B		0.26	0.44	1.71
	C3_01550C_B		0.25	0.47	1.84
	C7_01800C_B		0.32	0.83	2.62
	C5_04270C_B		0.23	0.49	2.11
	PGA4		0.36	0.67	1.87
	SIM1		0.26	0.47	1.79
	RPL4B		0.21	0.46	2.24
	RPS3		0.09	0.26	3.04
	PGI1		0.08	0.42	5.37
	PNG2		0.04	0.35	8.67
	C4_01520C_B		0.16	0.24	1.53
	GPH1		0.17	0.42	2.47
	FBA1		0.10	0.41	4.06
	RPL19A		0.16	0.31	1.87
	C2_06810C_B		0.08	0.15	1.84
	ALS3		0.03	0.10	3.27
	PIL1		0.10	0.20	1.97
	C2_08160C_B		0.05	0.16	3.19
	UTR2		0.08	0.28	3.63
	ILV5		0.02	0.24	13.59
	RPL16A		0.08	0.14	1.73
	RPS6A		0.13	0.26	2.05
	RPL6		0.05	0.25	5.09
	POR1		0.05	0.14	2.54
	TIF		0.08	0.16	1.98
	C4_04890C_B		0.08	0.13	1.64
	LSP1		0.10	0.15	1.53
	ILV2		0.07	0.19	2.66
	PMT1		0.19	0.35	1.81
	ACT1		0.03	0.19	5.87
	UGP1		0.11	0.27	2.37
	C2_08870C_B		0.12	0.20	1.69
	MET15		0.13	0.26	2.00
	LEU1		0.22	0.36	1.61
	HSP104		0.12	0.21	1.77
	MET3		0.09	0.23	2.66
	C4_00270W_B		0.08	0.14	1.72
	C1_06450C_B		0.09	0.21	2.18
	RPL32		0.08	0.18	2.21
	C1_03020C_B		0.05	0.15	2.71
	RPL12		0.04	0.16	3.86
	RPL2		0.08	0.12	1.61
	C3_05100C_B		0.05	0.14	2.68
	C1_11360W_B		0.04	0.14	3.72
	C1_01640W_B		0.02	0.13	7.28
	VRG4		0.08	0.13	1.57
	RPS13		0.07	0.13	1.79
	TSA1		0.02	0.15	8.51
	C1_05720W_A		0.02	0.18	10.14
	BGL2		0.12	0.18	1.48
	RPL43A		0.05	0.09	1.74
	CR_04510W_B		0.10	0.17	1.69
	MNT1		0.02	0.07	3.63
	C3_04910C_B		0.03	0.07	2.26
	SUR7		0.07	0.20	2.66

	C1_06580W_B	0.04	0.15	4.01
	RPL8B	0.03	0.16	4.99
	COX5	0.01	0.16	11.39
	OPT3	0.01	0.39	27.32
	C2_04460W_B	0.02	0.12	6.49
	RLI1	0.06	0.13	2.07
	C6_02260C_B	0.06	0.17	2.67
	CR_07630C_B	0.04	0.10	2.41
	C1_10870W_B	0.04	0.11	2.67
	RPS8A	0.04	0.11	2.98
	C7_00960W_B	0.04	0.13	3.57
	CSH3	0.03	0.11	3.53
	ENG1	0.07	0.18	2.58
	TUP1	0.01	0.13	9.52
	C1_11670W_B	0.03	0.08	2.55
	HHF1	0.04	0.15	3.64
	CAM1	0.01	0.05	3.63
	SAM2	0.03	0.07	2.08
	AQY1	0.04	0.08	2.09
	C3_00760W_B	0.04	0.11	2.89
	ILV3	0.02	0.09	4.99
	GLN1	0.02	0.10	5.53
	C4_04160W_B	0.04	0.07	1.86
	C6_00440C_B	0.05	0.16	2.94
	C4_03390W_B	0.05	0.09	1.93
	C1_03380W_B	0.02	0.08	4.55
	C7_00990W_B	0.02	0.03	1.63
	C4_05330C_A	0.05	0.11	2.17
	LAG1	0.03	0.06	1.86
	MEP2	0.06	0.09	1.47
	ERV29	0.03	0.08	2.36
	APE2	0.03	0.08	2.62
	ERG4	0.05	0.11	1.94
	ADE5,7	0.04	0.09	2.56
	AMO1	0.02	0.06	3.32
	C3_06910C_B	0.02	0.06	3.45
	HOM3	0.03	0.06	2.20
	CDR4	0.01	0.04	2.67
	SRB1	0.04	0.07	1.80
	C2_05410W_B	0.04	0.06	1.45
	ZUO1	0.01	0.09	6.02
	MYO5	0.02	0.06	3.32
	TRA1	0.03	0.05	1.52
	C4_02780W_A	0.02	0.04	2.46
	HOM2	0.04	0.07	1.62
	C7_03590C_A	0.02	0.04	2.40
	HSP78	0.01	0.04	3.12
	PDI1	0.01	0.10	6.90
	GAP2	0.02	0.15	8.22
	SEC23	0.02	0.04	2.10
	CR_01290C_B	0.01	0.02	1.73
	CR_06170W_A	0.01	0.07	5.13
	CHS3	0.01	0.02	1.56
	COX15	0.02	0.04	2.28
	FOX2	0.02	0.04	2.10
	C3_03410C_B	0.01	0.02	1.56
	STE24	0.01	0.02	1.73
	C5_02380W_A	0.01	0.02	1.56
	C2_09950W_B	0.02	0.06	3.32
	DUR1,2	0.02	0.04	2.10
	C1_14240W_B	0.02	0.04	2.10
	C1_11200W_B	0.04	0.07	1.80
	C4_05140C_B	0.02	0.04	2.10
	POM152	0.02	0.04	2.28
	STI1	0.01	0.04	2.67
	C2_05290C_B	0.01	0.02	1.73
	C7_02690C_A	0.01	0.02	1.56
	CDC46	0.01	0.02	1.56
**Downregulated**	FAS1	3.53	1.74	0.49
	CR_00640W_B	2.65	1.10	0.41
	CR_07050C_B	2.86	1.00	0.35
	URA2	2.83	1.00	0.35
	PYC2	1.41	0.58	0.41
	MSI3	0.69	0.33	0.48
	C1_05630C_A	0.37	0.11	0.29
	MET10	0.59	0.26	0.44
	ELF1	0.52	0.13	0.25
	CPA2	0.49	0.18	0.38
	RPA190	0.43	0.14	0.32
	C1_08090C_B	0.51	0.20	0.38
	CHO2	0.36	0.11	0.30
	MIS12	0.35	0.09	0.24
	NDE1	0.27	0.09	0.32
	RPO21	0.21	0.09	0.43
	C7_00570W_B	0.28	0.09	0.34
	C1_01590C_A	0.21	0.08	0.38
	C1_13780W_B	0.28	0.02	0.09

CR_10140W_A	0.19	0.00	0.00
TIF4631	0.30	0.11	0.37
MES1	0.27	0.10	0.38
GAP4	0.25	0.08	0.33
C5_04700C_B	0.27	0.09	0.31
C4_06110C_B	0.18	0.04	0.23
ZWF1	0.10	0.04	0.38
C1_14080W_A	0.15	0.02	0.16
RPG1A	0.22	0.00	0.00
C1_11660W_B	0.03	0.00	0.00
C2_05500W_B	0.01	0.00	0.00
CR_02860W_B	0.05	0.02	0.34
C7_01030C_B	0.12	0.02	0.15
SEC24	0.08	0.02	0.23
CR_05520W_B	0.16	0.02	0.15
COX2	0.06	0.02	0.42
C3_05370C_B	0.09	0.02	0.20
C2_03080W_B	0.04	0.00	0.00
C2_03820C_B	0.08	0.00	0.00
MTS1	0.02	0.00	0.00
ACH1	0.05	0.02	0.41
YCF1	0.11	0.04	0.38
C3_06100C_B	0.13	0.05	0.39
C1_05660C_A	0.16	0.00	0.00
AGC1	0.12	0.02	0.21
C1_00210C_A	0.06	0.02	0.32
GCV2	0.09	0.02	0.22
CR_04240C_A	0.07	0.00	0.00
STT3	0.07	0.00	0.00
C5_01210W_B	0.06	0.02	0.29
RPL10A	0.04	0.00	0.00
PHO88	0.04	0.00	0.00
ATP17	0.04	0.00	0.00
CDC68	0.11	0.02	0.21
C1_13060C_A	0.05	0.02	0.41
C3_01960C_B	0.05	0.02	0.38
CDC53	0.03	0.00	0.00
C2_00700W_A	0.06	0.02	0.32
C3_06970W_A	0.05	0.02	0.44
C3_06030W_B	0.13	0.00	0.00
SSH1	0.06	0.02	0.38
C7_00320C_B	0.10	0.02	0.22
SSN6	0.07	0.02	0.30
NAD1	0.06	0.00	0.00
C4_04870C_A	0.11	0.04	0.38
YHM2	0.01	0.00	0.00
CR_00780C_B	0.04	0.02	0.47
TNA1	0.03	0.00	0.00
C1_10680C_B	0.05	0.00	0.00
C2_02720W_A	0.12	0.00	0.00
C1_07690C_A	0.08	0.00	0.00
C6_03790C_B	0.01	0.00	0.00
DIP2	0.05	0.00	0.00
PIM1	0.07	0.00	0.00
C3_04390W_B	0.05	0.02	0.43
YHM1	0.04	0.00	0.00
C2_01340W_A	0.05	0.00	0.00
UTP22	0.07	0.00	0.00
ARG5,6	0.02	0.00	0.00
MSS116	0.03	0.00	0.00
C1_03790C_A	0.02	0.00	0.00
C3_05730C_B	0.04	0.00	0.00
C4_06730C_A	0.03	0.00	0.00
OST1	0.03	0.00	0.00
C3_04470W_B	0.03	0.00	0.00
C4_03360C_B	0.05	0.00	0.00
C7_00520W_A	0.07	0.00	0.00
FRE7	0.01	0.00	0.00
CR_06470W_A	0.02	0.00	0.00
CR_08000C_A	0.02	0.00	0.00
C7_03050W_B	0.04	0.00	0.00
CR_01110W_B	0.02	0.00	0.00
CR_00230W_B	0.06	0.00	0.00
C2_04870C_A	0.05	0.00	0.00
C1_03100W_A	0.02	0.00	0.00
C4_01720C_B	0.04	0.00	0.00
LEU4	0.05	0.00	0.00
CR_04170W_A	0.01	0.00	0.00
C2_00360C_A	0.08	0.00	0.00
CDC47	0.04	0.02	0.47
C1_02760W_B	0.04	0.00	0.00
CDC54	0.04	0.00	0.00
RET2	0.04	0.00	0.00
TIF3	0.04	0.00	0.00
RPP1A	0.04	0.00	0.00
HAS1	0.04	0.00	0.00
UBA2	0.04	0.00	0.00

MUTANT SECRETOMES

Protein	Alias	*crz1*			*gpr1gpa2*		
		Average Glu PSM (n=4)	Average GluLac PSM (n=4)	Fold Change GluLac/Glu PSM	Average Glu PSM (n=4)	Average GluLac PSM (n=4)	Fold Change GluLac/Glu PSM
MP65		18.17	17.72	0.98	15.45	20.22	1.31
C2_10030C_B	MP65	6.78	7.39	1.09	10.27	13.58	1.32
C3_01550C_B	TOS1	10.56	8.92	0.85	9.13	16.51	1.81
TOS1		11.35	12.47	1.10	9.08	15.28	1.68
SUN41		8.85	10.46	1.18	6.70	7.87	1.17
SCW11		3.87	5.98	1.54	3.14	6.64	2.12
CHT2		4.61	5.51	1.19	2.18	2.47	1.13
XOG1		1.87	1.45	0.78	7.65	n.d.	n.d.
ALS4		3.24	0.98	0.30	2.14	1.23	0.58
ALS2		2.95	1.96	0.67	2.71	n.d.	n.d.
SIM1		0.54	6.40	11.91	3.14	n.d.	n.d.
UTR2		1.37	0.47	0.34	2.66	n.d.	n.d.
BGL2		1.37	0.47	0.34	3.33	n.d.	n.d.
RBE1		1.91	1.96	1.03	1.62	n.d.	n.d.
C1_07030C_B	RBT4	1.61	1.03	0.64	2.81	n.d.	n.d.
ACT1		2.70	0.98	0.36	1.00	1.00	1.00
CR_10110W_B	CHT3	1.37	1.96	1.43	1.66	1.00	0.60
C6_04380W_B	ALS2	1.91	0.98	0.51	1.04	1.00	0.96
ECM33		n.d.	0.51	>0.51	1.14	1.23	1.08
PLB4.5		3.58	n.d.	<3.58	1.66	n.d.	n.d.
RBT4		1.61	0.51	0.32	2.18	n.d.	n.d.
C1_04180W_B	HTB2	1.33	0.98	0.74	0.67	4.17	6.25
C3_03280C_B	HTA3	2.17	0.51	0.24	1.14	1.23	1.08
DAG7		0.54	1.00	1.86	2.33	1.00	0.43
PHR2		0.54	0.98	1.83	1.14	0.10	0.09
C7_00860W_B	SSR1	2.74	1.00	0.36	1.04	0.10	0.10
C7_01560C_B	NUP	1.37	0.47	0.34	n.d.	n.d.	n.d.
YWP1		n.d.	0.98	>0.98	1.00	n.d.	n.d.
ENO1		1.00	2.05	2.05	n.d.	n.d.	n.d.
TDH3		0.10	1.03	10.26	n.d.	1.23	n.d.
C1_08380W_B	TEF2	0.10	0.98	9.82	n.d.	4.17	n.d.
ATC1		0.10	1.45	14.52	n.d.	n.d.	n.d.
ENG1		0.54	0.47	0.87	n.d.	n.d.	n.d.
PGA4		n.d.	n.d.	n.d.	1.00	0.10	0.10
C4_04470W_B	SAP10	n.d.	n.d.	n.d.	1.00	0.10	0.10
ALS3		n.d.	n.d.	n.d.	n.d.	n.d.	n.d.
C2_08870C_B	PIR1	0.54	0.10	0.19	n.d.	n.d.	n.d.
PGK1		0.10	0.51	5.13	n.d.	n.d.	n.d.
C4_06570C_B	PDC11	0.10	0.51	5.13	n.d.	n.d.	n.d.
C1_05960W_B	PGA45	0.10	0.47	4.69	n.d.	n.d.	n.d.
C7_02260W_B	LDG2	0.10	0.47	4.69	n.d.	n.d.	n.d.
C3_05130C_B	THI4	n.d.	n.d.	n.d.	1.00	4.17	4.17
